# Nomogram-based prediction of portal vein system thrombosis formation after splenectomy in patients with hepatolenticular degeneration

**DOI:** 10.3389/fmed.2023.1103223

**Published:** 2023-02-23

**Authors:** Zhou Zheng, Qingsheng Yu, Hui Peng, Long Huang, Wanzong Zhang, Yi Shen, Hui Feng, Wenshan Jing, Qi Zhang

**Affiliations:** ^1^The First Affiliated Hospital of Anhui University of Chinese Medicine, Hefei, Anhui, China; ^2^Institute of Chinese Medicine Surgery, Anhui Academy of Chinese Medicine, Hefei, Anhui, China

**Keywords:** hepatolenticular degeneration, splenectomy, portal vein system thrombosis, nomogram, predictive variables

## Abstract

**Objective:**

Splenectomy is a vital treatment method for hypersplenism with portal hypertension. However, portal venous system thrombosis (PVST) is a serious problem after splenectomy. Therefore, constructing an effective visual risk prediction model is important for preventing, diagnosing, and treating early PVST in hepatolenticular degeneration (HLD) surgical patients.

**Methods:**

Between January 2016 and December 2021, 309 HLD patients were selected. The data were split into a development set (215 cases from January 2016 to December 2019) and a validation set (94 cases from January 2019 to December 2021). Patients’ clinical characteristics and laboratory examinations were obtained from electronic medical record system, and PVST was diagnosed using Doppler ultrasound. Univariate and multivariate logistic regression analyses were used to establish the prediction model by variables filtered by LASSO regression, and a nomogram was drawn. The area under the curve (AUC) of receiver operating characteristic (ROC) curve and Hosmer–Lemeshow goodness-of-fit test were used to evaluate the differentiation and calibration of the model. Clinical net benefit was evaluated by using decision curve analysis (DCA). The 36-month survival of PVST was studied as well.

**Results:**

Seven predictive variables were screened out using LASSO regression analysis, including grade, POD14D-dimer (Postoperative day 14 D-dimer), POD7PLT (Postoperative day 7 platelet), PVD (portal vein diameter), PVV (portal vein velocity), PVF (portal vein flow), and SVD (splenic vein diameter). Multivariate logistic regression analysis revealed that all seven predictive variables had predictive values (*P* < 0.05). According to the prediction variables, the diagnosis model and predictive nomogram of PVST cases were constructed. The AUC under the ROC curve obtained from the prediction model was 0.812 (95% CI: 0.756–0.869) in the development set and 0.839 (95% CI: 0.756–0.921) in the validation set. Hosmer–Lemeshow goodness-of-fit test fitted well (*P* = 0.858 for development set; *P* = 0.137 for validation set). The nomogram model was found to be clinically useful by DCA. The 36-month survival rate of three sites of PVST was significantly different from that of one (*P* = 0.047) and two sites (*P* = 0.023).

**Conclusion:**

The proposed nomogram-based prediction model can predict postoperative PVST. Meanwhile, an earlier intervention should be performed on three sites of PVST.

## Introduction

Hepatolenticular degeneration (HLD) is a copper metabolic disorder caused by mutations in ATP7B gene encoding copper transporting ATPase (1). ATP7B protein is mainly expressed in the liver. The mutation of ATP7B gene leads to copper transport dysfunction and results in excessive deposition of copper in the liver, which further alters mitochondrial function in hepatic cells and causes damage to the lipid, protein, DNA, RNA, and other molecules, ultimately resulting in liver injury and liver steatosis. Copper deposition can also activate hepatic stellate cells and accelerate the progression of liver fibrosis (2). Statistically, there are 1/90 carriers of ATP7B mutation worldwide, and HLD prevalence is about 0.25/10,000∼4/10,000 (3, 4). The estimated prevalence of HLD in Europe has increased from 5 to 142 per million in the last 50 years. The prevalence of HLD in South Korea is 38.7 per million. In Japan, it is estimated to be 1.21/10,000 to 1.96/10,000 based on the mutation of ATP7B (5, 6). In a recent study, 1,533,370 people were investigated in Anhui Province, China, and nine cases of HLD were found, with an estimated incidence of 17.93 per million (7). The liver was found to be the most frequently involved and one of the earliest affected organs in the HLD population (onset may occur as young as 2 years old), and its main consequences are hepatitis, cirrhosis, and even liver failure (8).

However, about 35–45% of diagnosed HLD patients already had cirrhosis, whether the primary manifestation was liver injury, neuropsychiatric damage, or asymptomatic cases (9–11). Other major clinical features included malaise, loss of appetite, esophageal and gastric varicose veins, and even ascites, splenomegaly, as well as hypersplenism. Currently, the mainstays of HLD treatment are drugs, including penicillamine, sodium dimercaptopropyl sulfonate, and other complexing agents. However, these lifelong copper-repellent drugs have myelosuppression, decreasing peripheral blood cells. Besides, the increased destruction caused by splenomegaly and hypersplenism would also lead to the dilemma of the treatment (12). In 1993, Professor Yang Renmin and his colleagues at the HLD Diagnosis and Treatment Center of our hospital achieved a satisfactory effect during the therapy of HLD patients with splenomegaly and hypersplenism using strong anti-copper treatments before and after splenectomy (1). However, postoperative portal venous system thrombosis (PVST) formation is one of the most common complications, and the characteristic clinical manifestations are upper gastrointestinal bleeding, small intestinal ischemic necrosis, intractable ascites, jaundice, hepatic encephalopathy, and other serious damage that influence the survival and prognosis of the patients (13).

Therefore, early ultrasound examination plays a vital role in PVST prevention and treatment, which is conducive to reducing the mortality and disability rate caused by liver function deterioration and small intestine necrosis, etc. On the one hand, our study is focused on constructing a simple and effective risk model to screen out independent influencing factors of PVST and predict the risk of PVST in HLD patients after splenectomy. On the other hand, it is focused on analyzing the number of PVST sites to benefit the patients’ survival. Collectively, all these findings aim at early diagnosis, treatment, and improvement of prognosis in HLD patients after surgery.

## Materials and methods

### Study population and study design

A total of 309 HLD patients were selected from the Department of General Surgery of the First Affiliated Hospital of Anhui University of Chinese Medicine from January 2016 to December 2021. The patients’ data were divided into two parts: the patients from January 2016 to December 2019 were used as the development set (215 cases), and the others from January 2019 to December 2021 were used as the validation set (94 cases). The diagnostic criteria were determined as follows:

(1) HLD: (i) extraspinal symptoms and neuropsychiatric symptoms; (ii) liver damage with elevated serum transaminase and positive corneal of Kayser–Fleischer ring; (iii) serum ceruloplasmin < 200 mg/L and 24 h output volume of urine copper > 100 μg; and (iv) characteristic changes in liver and brain imaging (14);

(2) PVST: Portal vein thrombosis (trunk and intrahepatic branches), mesenteric vein thrombosis, and splenic vein thrombosis were demonstrated by hyperechoic or isoechoic filling in the cavity by Doppler ultrasound/CT/MRI, and venous diameter may be dilated in the acute phase (15).

(3) Splenomegaly grade: (i) mild degree (I): the lower boundary of the spleen was less than 3 cm below the costal arch when deep inspiration; (ii) moderate degree (II): the lower boundary of the spleen was 3 cm beyond the costal margin, but did not exceed the umbilical level and median abdominal line; (iii) severe degree (III): The lower boundary of the spleen extends beyond the umbilical level or the median abdominal line (giant spleen) (16).

(4) Hypersplenism grade: (i) mild: white blood cells (WBC) (3.0–4.0) × 10^9^, red blood cells (RBC) (2.5–3.5) × 10^12^, platelet (PLT) (7.0–10.0) × 10^9^; (ii) moderate: WBC (2.0–3.0) × 10^9^, RBC (1.5–2.5) × 10^12^, PLT (5.0–7.0) × 10^9^; (iii) severe: WBC < 2.0 × 10^9^, RBC < 1.5 × 10^12^, PLT < 5.0 × 10^9^ (16).

The inclusion criteria were set as follows:

(1) Splenomegaly and hypersplenism: WBC < (3.0–4.0) × 10^9^/L; RBC < (2.5–3.5) × 10^12^/L; PLT < (7.0–10.0) × 10^9^/L;

(2) HLD patients diagnosed with cirrhosis and portal hypertension who underwent splenectomy according to clinical, B-ultrasound, CT, or MRI examination;

(3) Bone marrow puncture demonstrated signs of myelodysplasia;

(4) The Child–Pugh class is A or B.

The exclusion criteria were set as follows:

(1) Cirrhosis complicated with blood system diseases and immune system diseases;

(2) Patients who had alcoholic hepatitis, schistosomiasis hepatitis and other types of liver disease that induce splenomegaly and hypersplenism;

(3) Patients with cardiac and renal insufficiency or complicated gastrointestinal tumor;

(4) Patients who underwent transjugular intrahepatic portal shunt (TIPS) or partial splenic embolization (PSE) before.

### Treatment

Patients with liver dysfunction were given liver protection treatment, vitamin supplementation, and anemia correction before surgery, especially for Child–Pugh class C, which should be adjusted to class B. Open splenectomy was used in all cases. The splenic colonic and gastric splenic ligaments were separated, and the splenic artery was fully exposed and ligated under unambiguous vision. Next, the second- and third-grade branch vessels of the spleen were dissected and ligated one by one. After that, the splenic ligament was dissociated sharply, and the spleen was completely removed. For patients with pericardia varices, the esophageal branches, lateral branches, and inferior diaphragmatic branches about 5 cm in the lower esophagus were ligated and severed. Finally, drainage tubes were placed, followed by layer and layer suture. Postoperative anti-infection, liver protection and other support treatments were given.

### The collection of clinical features and laboratory indicators

The included demographic features were age, sex, and Child–Pugh classification (scores of 5–6, 7–9, and 10–15 for classes A, B, and C, respectively). Method of surgery, whether anatomic splenectomy was performed, BMI at admission: BMI = weight (kg)/height (m^2^), diabetes mellitus, hypertension, smoking history (one or more cigarettes daily for 6 months), duration of surgery, intraoperative bleeding and splenomegaly grade. Patients fasted for 8–12 h, and 5 mL of venous blood was extracted at 8 am before the procedure. Automatic hemacytometer (XN-9000, Sysmex Corporation, Japan) was used to detect the level of WBC, RBC, hemoglobin (HGB), and hematocrit (HCT) by counter method. Activated partial thromboplastin time (APTT), prothrombin time (PT), and fibrinogen (FIG) were determined using the coagulation method, and D-dimer was determined using immunoturbidimetry. Automatic biochemical analyzer (7600-010, Hitachi, Ltd. Japan) was employed to detect the level of alanine transaminase (ALT), while aspartate transaminase (AST) was determined using the rate method. Albumin (ALB) concentration was measured using bromocresol green method, while total bilirubin (TBIL) was measured using the diazo method. Blood urea nitrogen (BUN) level was ascertained using HMMPS method, and serum creatinine (CER) was determined using the uric acid method. The dynamics of blood flow [portal vein diameter (PVD), splenic vein diameter (SVD), portal vein flow (PVF), and portal vein velocity PVV)] were determined using color Doppler ultrasound (ACUSON Antares, Siemens AG, Germany) with 5.0 MHz wide-screen concave array probe.

### Statistical analysis

Statistical Package for Social Sciences 26.0 (SPSS Inc, Chicago, USA) and R (Version 4.1.3) were used to analyze and plot the data. Normal data were expressed in terms of mean ± standard deviation, while the median (quartile) [M (P25, P75)] was adopted for the non-normal data. Both groups used the independent *T*-test or rank-sum test (Mann–Whitney U). Categorical data were expressed as percentages, and the chi-squared test was selected for intergroup comparison.

The least absolute shrinkage and selection operator (LASSO) was used to screen primary data using R “glmnet” package in development set. Single- and multi-factor logistic regression analyses were performed to filter the influence factors of PVST, and regression equations were constructed for meaningful variables. A nomogram was created using R packages “regplot,” and the differentiation of nomogram was determined by the area under the curve (AUC) of the receiver operating characteristic (ROC) curve using the “pROC” package. Calibration was performed using Hosemer–Lemeshow with “ResourceSelection” package, and decision curve analysis (DCA) obtained by R “rmda” package was used to evaluate the clinical utility of the nomogram model. The external data were endorsed by the validation set. The endpoint was defined as the time from the date of surgery to the date of death; otherwise, it was defined as censored data. Kaplan–Meier calculation was performed by R “survminer” and “survival” packages and compared with log-rank test, and data were plotted by “ggplot” packages.

## Results

### The principle of splenectomy and portal vein system thrombosis

**FIGURE 1:**
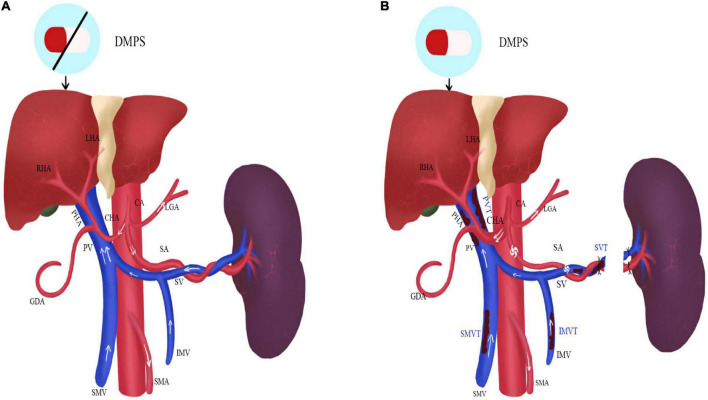
The status of the portal vein system thrombosis before and after operation in HLD patients. **(A)** Before operation; **(B)** after operation; DMPS, 2,3-Dimercaptopropanesulfonic Sodium; LHA, left hepatic artery; RHA, right hepatic artery; PHA, proper hepatic artery; CHA, common hepatic artery; GDA, gastroduodenal artery; CA, celiac artery; LGA, left gastric artery; SA, splenic artery; SMA, superior mesenteric artery; PV, portal vein; SMV, superior mesenteric vein; IMV, inferior mesenteric vein; PVT, portal venous thrombosis; SVT, splenic vein thrombosis; SMVT, superior mesenteric vein thrombosis; IMVT, inferior mesenteric vein thrombosis.

The long-term pathological changes of hepatolenticular cirrhosis, such as higher hepatic vascular resistance, higher visceral hemodynamics, and relatively lower splenic vascular resistance, cause spleen enlargement and hyperactivity. It results in an increase in the splenic artery diameter and blood flow velocity and a decrease in blood cell count. Currently, bone marrow suppressive copper-repellent drugs (such as penicillamine and 2,3-dimercaptopropanesulfonic sodium) exacerbate blood cell depletion, increasing the risk of bleeding and infection and making long-term copper removers impossible (Figure 1A).

After splenectomy, blood cell count rises rapidly, especially platelets. It makes blood appear hypercoagulable and consequently increases the risk of thrombosis. Moreover, since portal venous blood flow decline rapidly, the reduction of portal vein velocity and diameter and the vortex formation of the splenic vein stump also lead to thrombosis. However, the thickened hepatic artery increases blood flow and improves liver function afterward. The copper-repellent drugs can be carried out for a life-long time due to the recovery of blood cell count (Figure 1B).

### Demographic characteristics

**FIGURE 2:**
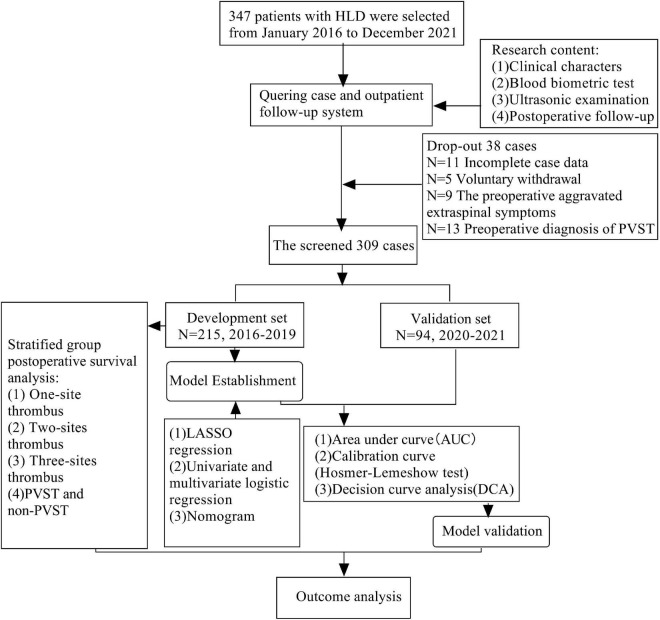
Flow chart of selection and analysis of development set and validation set of HLD patients.

Figure 2 reveals that 309 patients were included in this research, including 215 in the development set and 94 in the validation set. Age, gender, Child–Pugh classification, surgical method, anatomic splenectomy, BMI, diabetes, hypertension, smoking history, operation time, intraoperative bleeding, splenomegaly grade, levels of WBC, RBC, HGB, PLT, HCT, APTT, PT, FIG, D-dimer, ALT, AST, ALB, TBIL, BUN, CER, PVD, SVD, PVV, and PVF revealed no statistical significance between two groups (*P* < 0.05), as evident from Table 1 and Supplementary materials 1, 2.

**TABLE 1:** Feature comparison of development set and validation set.

Clinical features	Development set	Validation set	*t/z/*χ *2*	*P*-value
Age (years)	28.00 (21.00 34.00)	29.00 (21.00 36.00)	−0.139	0.890
Gender [Male/Female, n (%)]	110 (51.16)/105 (48.84)	53 (56.38)/41 (43.62)	0.715	0.398
Child-Pugh classification [A/B n (%)]	119 (55.35)/96 (44.65)	58 (61.70)/36 (38.30)	1.079	0.299
Surgery Method [n (%)]			1.092	0.296
Splenectomy	192 (89.30)	80 (85.11)		
Splenectomy combined with esophagogastric devascularization	23 (10.70)	14 (14.89)		
Anatomic splenectomy [Y/N, n (%)]	121 (56.28)/94 (43.72)	50 (53.19)/44 (46.81)	0.252	0.615
BMI [n (%)]			1.871	0.392
<18.5 kg/m^2^	21 (9.77)	14 (14.89)		
18.5–24.9 kg/m^2^	68 (31.63)	26 (27.66)		
≥ 25 kg/m^2^	126 (58.60)	54 (57.45)		
Diabetes [Y/N, n (%)]	41 (19.07)/174 (80.93)	17 (18.09)/77 (81.91)	0.042	0.838
Hypertension [Y/N, n (%)]	34 (15.81)/181 (84.19)	17 (18.09)/77 (81.91)	0.245	0.621
Smoking history [Y/N, n (%)]	29 (13.49)/186 (86.51)	19 (20.21)/75 (79.79)	2.254	0.133
Ascites [Y/N, n (%)]	72 (33.49)/143 (66.51)	34 (36.17)/60 (63.83‘)	0.209	0.648
Thickness of spleen (mm)	4.96 (4.44 5.55)	5.02 (4.59 5.65)	−0.622	0.534
Splenomegaly grade [n (%)]			−0.500	0.617
Mild (I)	55 (25.58)	34 (36.17)		
Moderate (II)	91 (42.33)	25 (26.60)		
Severe (III)	69 (32.09)	35 (37.23)		
**D-dimer (mg/L)**				
Before	0.66 (0.55 0.78)	0.65 (0.58 0.69)	−1.299	0.194
POD14	15.34 (12.99 18.16)	14.52 (12.31 17.07)	−0.716	0.152
INR	1.35 ± 0.14	1.30 ± 0.17	1.896	0.060
APTT (s)	45.07 ± 8.52	44.87 ± 3.67	0.289	0.773
PT (s)	14.90 (13.80 16.11)	14.71 (13.30 16.20)	−0.595	0.552
FIB (g/L)	1.68 ± 0.21	1.67 ± 0.31	0.326	0.745
WBC (× 10^9^/L)	2.66 ± 0.84	2.58 ± 0.38	1.176	0.240
RBC (× 10^12^/L)	3.18 ± 0.49	3.24 ± 0.25	−1.354	0.177
**PLT (× 10^9^/L)**				
Before	57.55 ± 8.00	59.19 ± 7.28	−1.705	0.089
POD7	470.32 ± 35.67	471.11 ± 28.68	−0.206	0.837
HCT (%)	34.53 ± 2.51	34.22 ± 5.71	0.504	0.615
AST (U/L)	34.75 ± 7.07	34.32 ± 6.16	0.544	0.587
ALT (U/L)	30.02 ± 11.97	29.36 ± 6.24	0.632	0.528
ALB (g/L)	37.68 ± 4.52	37.36 ± 4.05	0.591	0.555
TBIL (μ mmol/L)	27.54 ± 4.79	26.67 ± 3.26	−0.261	0.794
BUN (mmol/L)	6.66 ± 0.50	6.36 ± 1.69	1.668	0.098
CER (u mol/L)	84.33 (79.81 88.88)	83.47 (74.89 92.37)	−0.498	0.618
Operation time (min)	213.90 ± 7.43	217.63 ± 47.41	−0.759	0.450
Intraoperative bleeding (mL)	220.64 ± 8.36	222.57 ± 41.49	−0.448	0.655
PVD (mm)	14.18 ± 2.54	13.81 ± 3.16	0.983	0.327
SVD (mm)	9.58 ± 2.29	9.80 ± 2.40	−0.752	0.453
PVV (cm/s)	23.13 ± 1.91	23.15 ± 3.05	−0.040	0.968
PVF (ml/min)	907.18 ± 37.50	901.74 ± 40.34	1.145	0.253

The data are expressed as mean ± standard deviation, median (interquartile range) values, or ratio of patients. SPD, splenectomy with portal devascularization; Before, preoperative day; POD, postoperative day; BMI, body mass index; ALB, albumin; AST, aspartate transaminase; ALT, alanine transaminase; TBIL, total bilirubin; WBC, white blood cells; HB, hemoglobin; HCT, hematocrit; APTT, activated partial thromboplastin time; PT, prothrombin time; INR, international standardized ratio; FIB, fibrinogen; PLT, perioperative platelet count; BUN, blood urea nitrogen; CER, serum creatinine; PVD, portal vein diameter; SVD, splenic vein diameter; PVV, portal vein velocity; PVF, portal vein flow.

### The screening variables of LASSO

**FIGURE 3:**
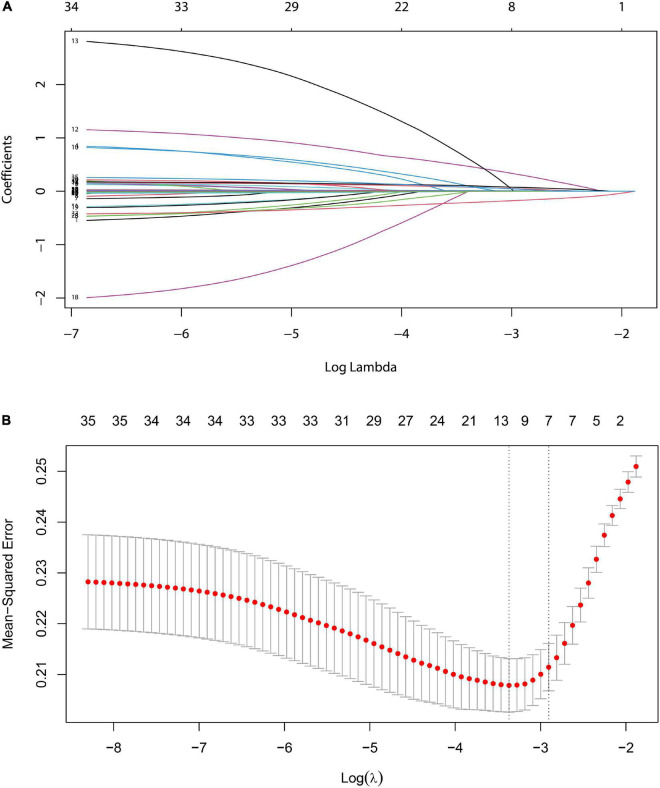
LASSO logistic regression model for clinical feature selection. **(A)** Distribution diagram of the model was drawn for logarithmic (lambda) sequences under different penalty coefficients. **(B)** 10-fold cross-validation: The first vertical line is the minimum error, and the second line is the cross-validation error of 1-time minimum standard deviation.

Based on the demography, biochemical, and imaging examination of patients in the development set, seven non-zero coefficient predictors were screened out between 33 variables using LASSO regression analysis (Figure 3). A vertical line was drawn at the λ minimum value (λ = 0.024) and λ minimum value of 1 SE (λ = 0.055), respectively. When log (λ) = –2.905, seven non-zero coefficient predictive variables were screened, for which LASSO regression constituted the most appropriate penalized linear model with a shrinkage penalty. The predictive variables for screening included splenomegaly grade, POD14D-dimer, POD7PLT, preoperative indicators of PVD, SVD, PVV, and PVF.

### The construction of prediction model

**FIGURE 4:**
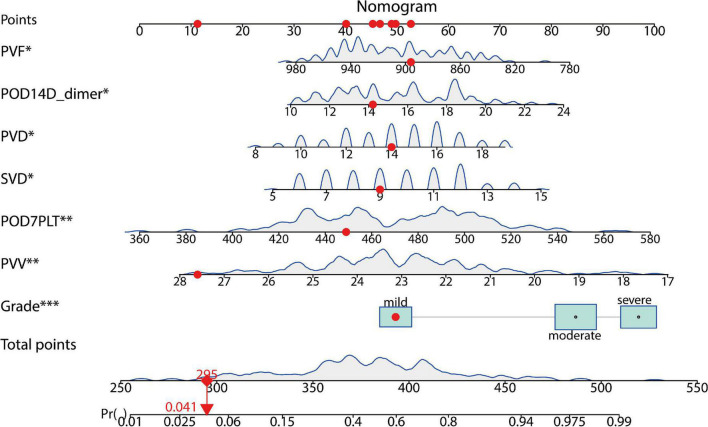
A predictive model for the risk of PVST in HLD patients. The nomogram gives every predictive variable a score on the point scale. A total score was calculated by summing the points from each variable, marking the estimated probability of PVST occurrence in HLD patients after surgery. For example, the red dots display a 4% risk of thrombosis in the first patient. POD, postoperative day; PVD, portal vein diameter; SVD, splenic vein diameter; PVV, portal vein velocity; PVF, portal vein flow. The “*”, “**”, and “***” symbols mean *P* < 0.05, *P* < 0.01, and *P* < 0.001, respectively.

Taking PVST occurrence in HLD patients as the dependent variable (non-PVST = 0, PVST = 1), univariate and multivariate logistic regressions were used to establish the clinical prediction model by the seven selected variables of LASSO regression analysis. The results indicated that splenomegaly grade, POD14D-dimer, POD7PLT, preoperative indicators of PVD, SVD, PVV, and PVF were the influencing factors of PVST in HLD patients *(P* < 0.05). The formula was as follows: Logit *P* = 0.862 + 1.263 Splenomegaly grade (Moderate vs. Mild) + 1.704 Splenomegaly grade (Severe vs. Mild) + 0.137 POD14D-dimer (mg/L) + 0.016 POD7PLT (× 10^9^/L) + 0.159 PVD + 0.188 SVD–0.311 PVV- 0.010 PVF [AIC (min): 242.05], as displayed in Table 2. The nomogram was drawn based on the predicted variables (Figure 4).

**TABLE 2:** Univariate and multivariate logistic regressions of influencing factors of portal venous system thrombosis (PVST) in the development set.

Types	Univariate analysis		Multivariate analysis	
	OR (95%CI)	*P*	OR (95%CI)	*P*
Splenomegaly grade [n (%)]	–	< 0.001	–	0.002
Mild (I)	Reference	–	Reference	–
Moderate (II)	3.940 (1.867 ∼ 8.313)	< 0.001	3.537 (1.496 ∼ 8.362)	0.004
Severe (III)	4.200 (1.919 ∼ 9.192)	< 0.001	5.498 (2.129 ∼ 14.196)	< 0.001
POD14D-dimer (mg/L)	1.162 (1.056 ∼ 1.279)	0.002	1.147 (1.021 ∼ 1.288)	0.021
POD7PLT (× 10^9^/L)	1.016 (1.007 ∼ 1.024)	< 0.001	1.016 (1.006 ∼ 1.027)	0.002
PVD (mm)	1.235 (1.101 ∼ 1.385)	< 0.001	1.172 (1.022 ∼ 1.345)	0.023
SVD (mm)	1.150 (1.020 ∼ 1.297)	0.022	1.207 (1.040 ∼ 1.401)	0.013
PVV (cm/s)	0.699 (0.592 ∼ 0.825)	< 0.001	0.733 (0.608 ∼ 0.883)	0.001
PVF (ml/min)	0.988 (0.980 ∼ 0.995)	0.001	0.990 (0.981 ∼ 0.999)	0.041

POD, postoperative day; OR, odds ratio; CI, confidence interval; PVD, portal vein diameter; SVD, splenic vein diameter; PVV, portal vein velocity, PVF, portal vein flow.

### Validation of prediction models

**FIGURE 5:**
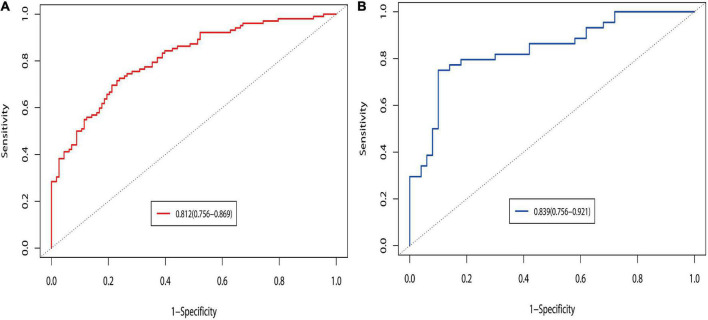
Discrimination of ROC curves in the development **(A)** and validation **(B)** sets. The prediction probability was calculated based on the development set model, and the discrimination of the model was evaluated by ROC curves under development and validation sets. Logit *P* = 0.862 + 1.263 Splenomegaly grade (Moderate vs. Mild) + 1.704 Splenomegaly grade (Severe vs. Mild) + 0.137 POD14D-dimer (mg/L) + 0.016 POD7PLT (× 109/L) + 0.159 PVD + 0.188 SVD-0.311 PVV- 0.010 PVF. Probability of prediction = exp (Logit P)/[1 + exp (Logit P)].

**FIGURE 6:**
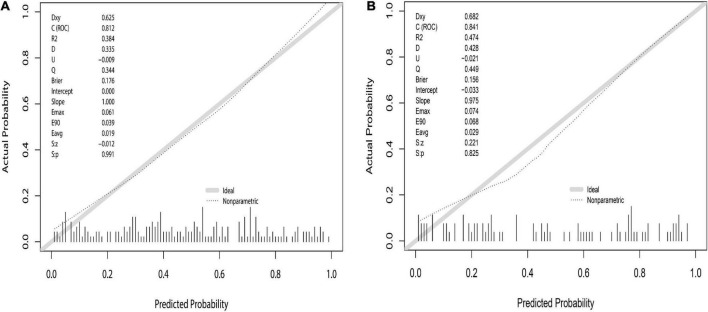
Nomogram calibration curve to predict the risk of PVST in HLD patients in the development **(A)** and validation **(B)** sets. The calibrations of development and validation sets are verified based on Hosmer–Lemeshow goodness-of-fit test, where *X*-axis represents the probability of nomogram prediction of PVST, and *Y*-axis represents the actual incidence of PVST.

The prediction model was validated based on the differentiation and calibration of the model. The differentiation of the prediction model was evaluated by drawing the ROC curve with AUC calculations to predict PVST occurrence in HLD patients following the procedure. The AUC results of the development set (0.812, 95% CI: 0.756–0.869, Figure 5A) and the validation set (0.839, 95% CI: 0.756–0.921, Figure 5B) indicated that the prediction model had good discriminant ability. Meanwhile, the Hosmer–Lemeshow goodness-of-fit test (development set *P* = 0.856, Figure 6A; validation set *P* = 0.137, Figure 6B) revealed that the predicted probability of the model is consistent with the actual probability, which demonstrates that it has a good calibration degree. Collectively, nomogram models displayed better predicting ability.

### The clinical decision curve

**FIGURE 7:**
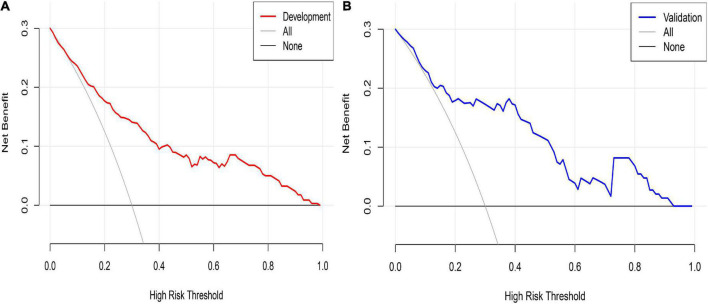
Nomogram decision curve analysis for PVST prediction in HLD patients in the development **(A)** and validation **(B)** sets. The *Y*-axis of development and validation sets represents the net benefit, calculated as the true incidence of PVST minus the false-positive rate (true positive rate), the red and blue lines represent the nomogram prediction probability of the development and validation sets, and the gray line represents the extremes of intervention and non-intervention.

Decision curve analysis was used to evaluate the clinical validity of the prediction model. The DCA of the postoperative PVST risk nomogram of HLD patients is revealed in Figure 7. The results demonstrated that the development set in the range of 15–99% and validation set in the range of 18–96% had better net benefits, which were higher than those of the prediction model with all measures taken (gray slash line) and no measures taken (gray horizontal line) for undiagnosed PVST; the implementation of intervention in this range is more favorable.

### Survival of portal venous system thrombosis after the operation

**FIGURE 8:**
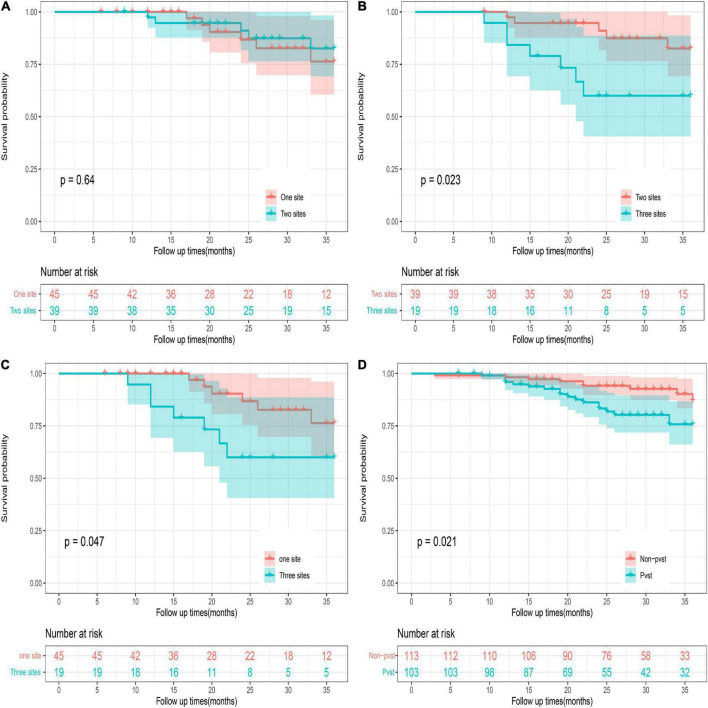
36-month survival analysis of the patients with different numbers of sites of thrombosis. PVST patients represented portal vein thrombosis (left and right branches and main trunk) or/and splenic vein thrombosis or/and mesenteric thrombosis. One site represents portal vein thrombosis (left and right branches and main trunk) or splenic vein thrombosis, or mesenteric thrombosis. Two sites: portal vein thrombosis (left and right branches and main trunk) and splenic vein thrombosis; portal vein thrombosis (left and right branches and main trunk) and mesenteric thrombosis; splenic vein thrombosis and mesenteric thrombosis. Three sites: portal vein thrombosis (left and right branches and main trunk) and splenic vein thrombosis + mesenteric thrombosis. Survival of patients with PVST after surgery: **(A)** One site versus two sites. **(B)** Two sites versus three sites. **(C)** One site versus three sites. **(D)** PVST versus non-PVST.

Portal venous system thrombosis after Wilson’s disease surgery was classified into portal vein thrombosis (left and right branches and main trunk), splenic vein thrombosis, and mesenteric thrombosis. PVST group was stratified into three groups (one-, two-, and three-site thrombus groups), and others were part of non-PVST group. The study of survival of postoperative patients at 36 months suggested that the death of the three groups did not exceed the median survival time, and the 36-month cumulative survival rate was 76.3, 82.5, 60.0, and 75.8%, respectively. There was no significant difference in survival rates between the one- and two-site thrombus groups (log-rank = 0.222; *P* = 0.640). A statistical difference could be highlighted between the one- versus three-site and two- versus three-site thrombus groups (log-rank = 3.928; *P* = 0.023, log-rank = 5.146; *P* = 0.047). There was obvious difference between the PVST and the non-PVST groups (log-rank = 5.337; *P* = 0.021), suggesting a need for early and positive intervention when PVST occurs, especially at three sites (Figure 8 and Supplementary materials 3–6).

## Discussion

Portal hypertension of cirrhosis is a potentially deadly complication of chronic liver disease. Currently, surgical treatments, including TIPS, PSE, splenectomy, and liver transplantation, are the main curative treatments for these populations (17–20). However, in the context of portal hypertension caused by HLD liver disease, the operative treatment aims to solve leukopenia and thrombocytopenia caused by hypersplenism to meet the life-long copper removal treatment; thus, TIPS surgery is unrecommended. The management strategy for updated guidelines for Wilson’s disease 2022 is primarily liver transplantation (12). This does not mean that a liver transplant should be performed once portal hypertension occurs (21). Studies have demonstrated that the mortality of portal hypertension hemorrhage is positively correlated with the severity of primary liver disease (Child–Pugh class A: < 10%, class B: 5–20%, class C: > 50%). Notably, the one-year survival rate of patients with Child–Pugh class A after variceal hemorrhage alone was equal to or even better than that of liver transplantation recipients (22, 23). Owing to adverse factors such as the shortage of liver donors, perplexing operation, high cost, complications related to liver transplantation, life-long immunosuppressive therapy, and recurrence of primary disease, liver transplantation is regarded as an alternative therapy for liver failure (end-stage liver disease) (24–26); Studies have evidenced that splenic embolism could improve leukocytes and platelets in the short term, but the long-term effect remains ambiguous. Moreover, the presence of extensive adhesion around the spleen greatly did not increase the surgical difficulty but was reported to harm patients and added to the treatment costs (27). Such an approach has only been utilized for class C cases, currently. Since HLD is an inherited metabolic disease, anti-copper therapies are essential for long-term survival. Currently, bone marrow suppression of copper-removal drugs, coupled with hypersplenism, can aggravate thrombocytopenia and leukopenia (28). Therefore, the main purpose of splenectomy is to restore normal blood cells to meet and ensure life-long copper-removal treatment.

However, the risk of PVST will increase due to the rapid recovery of blood cells (especially platelets), as well as the large splenic vein detachment and the formation of a local “whirlpool” following splenectomy. Some studies have indicated that the incidence of PVST in patients with portal hypertension under a natural state is about 0.6–2.1%. Postoperatively, it increased to 18.9–57.0% (29, 30). The presence of PVST not only aggravates liver damage and induces intestinal necrosis but also causes varicose hemorrhage again. Therefore, it is very important to predict the risk of PVST following splenectomy for HLD patients with cirrhosis and take effective interventions timely for these high-risk patients. As one of the intuitively displayed graphs in mathematical models, a nomogram can predict specific end points by combining multiple influencing factors. In addition, the nomogram has become a reliable and convenient tool for quantifying risk factors due to its ability to provide personalized assessments, thus facilitating disease management and clinical decision-making (31). Our study analyzed the clinical characteristics, hematology, and imaging examinations of PVST patients after splenectomy for HLD combined with cirrhosis and portal hypertension. Using LASSO, univariate, and multivariate regression analyses, we finally found that splenomegaly grade, POD14D-Dimer, POD7-PLT, and preoperative indexes of PVD, SVD, PVF, and PVV were the influencing factors of PVST, and then, a nomogram was constructed. A recent study has suggested that the greater the degree of spleen enlargement in patients with portal hypertension, the more likely the formation of PVST after splenectomy (32). Pietrabissa et al. noted that spleen weight was the only significant factor predictive of postoperative thrombosis. The combination of splenomegaly and an elevated preoperative platelet count was associated with a 75% incidence of this complication (33). Péré et al. reported that when the spleen weight was estimated to be greater than 500 g by CT on admission, active reexamination should be performed 5 days after surgery to exclude PVST formation (34). Our study depicted that the incidence of PVST after splenectomy is associated with splenomegaly grade (*P* = 0.002). The larger the spleen, the more severe the reduction of blood flow through the splenic vein into the portal vein system. Owing to an apparent descending of portal vein pressure after operation in the short term, a sudden decrease of blood flow into the portal vein system may appear, and thus, the incidence of PVST is likely to occur.

The postoperative imbalances of the blood coagulation system and the rise of platelet are also high-risk factors of PVST. D-dimer is a degradation product of cross-linked fibrin by factor XIII, which is a marker of activated coagulation and fibrinolysis. For patients with secondary fibrinolysis, the level of D-dimer is elevated (35). A recent study reported no statistical difference in D-dimer level between the patients in the thrombus group and the non-thrombus group preoperatively. However, after splenectomy, the levels of D-dimer displayed a significant difference between the two groups, and the specificity and sensitivity of predicting PVST were 76.9 and 83.5%, respectively, indicating that D-dimer can be used as a monitoring indicator of PVST formation (36). D-dimer was a risk factor for PVST 14 days after surgery in our study (*P* = 0.021; OR = 1.147; CI: 1.021–1.288). Thrombocytopenia in patients with cirrhosis has historically been attributed to hypersplenism because of portal hypertension. Post-splenectomy reactive thrombocytosis may occur in a short time due to resolution of hypersplenism. A previous study has shown that platelets in most postoperative patients begin to increase within 24 h, reaching a peak at 1–2 weeks, and return to normal after 4 weeks (37). These findings agree with our study that a significant difference in PLT level was observed between the two groups 7 days after the operation (*P* < 0.001). Multifactorial analysis suggested that PLT was a risk factor for PVST formation 7 days after the operation (*P* = 0.002; OR = 1.016; CI: 1.006–1.027). The reason may be that post-splenectomy platelet count rises rapidly in the short term, with a high blood coagulation state, especially for the large-volume-motivated new platelets releasing more vascular active substances (*P*-selection), thereby enhancing platelet adhesion and aggregation. Consequently, the possibility of thrombosis formation will be increased because it can release more precursor substances of thrombosis, such as alkane. The blood clot size was positively correlated with plasma *P*-selection level in two previous studies (29, 38). However, in clinical practice, not all PLT increases lead to PVST formation after surgery. We speculated that PLT level is not the only factor that affects postoperative thrombosis, and its importance warrants further investigation.

Postoperative hemodynamic alternations were considered the most important factor related to PVST formation. In the stage of HLD cirrhosis, the obstruction and resistance of portal vein blood flow are elevated, resulting in the increased portal and splenic vein pressure, compensatory splenic vein widening, and splenic congestive enlargement. The enlarged spleen would increase portal venous blood flow (39). After splenectomy, the splenic venous blood flow can be reduced by about 20–40%, which causes blood stasis. In addition, the formation of a local “vortex” after splenic vein dissection can also slow down the blood flow and lead to thrombosis (40). On the other hand, since vascular intima smooth muscle hyperplasia, muscle fiber thickening, and inflammatory cell infiltration had existed under the long-term hypertension of splenic and portal vein, the initiation of the coagulation system could occur (41). Senzolo et al. remarked that the remaining splenic vein formed a larger blind end following ligation of the widened splenic vein. The hematic stasis in it was more likely to promote thrombosis, which could then spread to the portal vein and superior mesenteric vein (42). The same results were obtained in this study: preoperative PVD, SVD, PVV, and PVF were the influencing factors of PVST (*P* = 0.023, OR = 1.172, CI: 1.022–1.345; *P* = 0.013, OR = 1.207, CI: 1.040–1.401; *P* = 0.001, OR = 0.733, CI: 0.608–0.883; *P* = 0.041, OR = 0.990, CI: 0.981–0.999).

Survival analysis between PVST and non-PVST groups depicted no significant difference at 36 months of follow-up from a retrospective study by Dong et al. (43). According to Shengjing classification of PVST. Song et al. found that the higher the thrombosis level, the wider the range of thrombosis involvement. During the follow-up process, subgroup analysis revealed that the overall survival rate of type I and II groups was statistically different from that of type III, while no difference was found between type I and type II groups (44). In our study, we analyzed cumulative survival at 36 months in each group according to the number of thrombotic sites. The results showed no differences between PVST and non-PVST groups (*P* = 0.021). Subgroup analysis demonstrated no difference between the one- and two-site thrombus groups (*P* = 0.64). Compared with the three-site thrombosis group, the difference between one site and two sites was statistically significant (*P* = 0.023; *P* = 0.047). All these findings suggest that for HLD patients with portal hypertension, compared to patients without venous thrombosis or limited local thrombosis after splenectomy, the wider range of PVST requires close attention and early intervention. Timely detection, severity evaluation, and treatment such as anti-coagulant intervention can contribute to the recovery of liver function and reduce fatal complications, ultimately benefiting long-term or even life-long anti-copper therapy for HLD patients.

Compared with the traditional diagnostic model, seven influencing factors of PVST were screened in this study. Finally, the established model was verified internally and externally. The AUC of the development and the validation sets were 0.812 and 0.839, respectively, while the calibration degree (*P* = 0.519; *P* = 0.137) indicated the robustness of the model. The DCA plots reveal a good favorable clinical net benefit of the nomogram. However, there are some non-negligible limitations in the current study. First, since this study was retrospective and external validation was conducted in different time periods, a large sample and multi-center were required. Second, the molecular mechanism of portal vein thrombosis remains unknown and needs further study in animal and cell experiments. Third, it can be considered that continuous variables can be classified and transformed, and then the optimal scale regression method can be used for screening, which is more convenient for the clinical application of the nomogram prediction model.

## Conclusion

Based on the nomogram, we have established a risk prediction model for PVST after splenectomy in HLD patients combined with cirrhosis and portal hypertension. Splenomegaly grade, POD14D-dimer, POD7PLT, preoperative indexes of PVD, SVD, PVV, and PVF were the influencing factors of PVST formation. The ROC, calibration, differentiation, and DCA of development and validation sets show that the established model has good prediction ability, aiding clinicians in diagnosis and decision-making. On the other hand, we carried out a stratified analysis of the number of PVST ranges after surgery. Patients with extensive PVST should maintain high vigilance. Effective prevention and treatment measures should be taken to reduce the risk of postoperative death by guaranteeing long-term copper removal for HLD patients.

## Data availability statement

The original contributions presented in this study are included in this article/Supplementary material, further inquiries can be directed to the corresponding author.

## Ethics statement

This studies involving human participants were reviewed and approved by the Ethics Committee of The First Affiliated Hospital of Anhui University of Chinese Medicine (Batch number: 2019AH-32), and Clinical trial Registration Number: ChiCTR2000034137. The study was carried out in accordance with the ethical principles laid down in the Declaration of Helsinki. Written informed consent to participate in this study was provided by the participants’ legal guardian/next of kin.

## Author contributions

QY contributed to the conceptual design of the study. HP, WZ, HF, and WJ contributed to data collection and analysis. LH, YS, and QZ were responsible for the treatments of the patients. ZZ interpreted and drafted the manuscript. All authors have read and approved this submitted version.
